# Association of B7-H4, PD-L1, and tumor infiltrating lymphocytes with outcomes in breast cancer

**DOI:** 10.1038/s41523-018-0095-1

**Published:** 2018-12-10

**Authors:** Mehmet Altan, Kelley M. Kidwell, Vasiliki Pelekanou, Daniel E. Carvajal-Hausdorf, Kurt A. Schalper, Maria I. Toki, Dafydd G. Thomas, Michael S. Sabel, Daniel F. Hayes, David L. Rimm

**Affiliations:** 1 Section of Medical Oncology, Yale School of Medicine, New Haven, CT USA; 2 Department of Thoracic/Head & Neck Medical Oncology, The University of Texas MD Anderson Cancer Center, Houston, TX USA; 3 Department of Biostatistics, School of Public Health, University of Michigan, Ann Arbor, MI USA; 4 Breast Oncology Program, University of Michigan Comprehensive Cancer Center, Ann Arbor, MI USA; 5 Department of Pathology, Yale School of Medicine, New Haven, CT USA; 6 Anatomic Pathology, Clinica Alemana-Facultad de Medicina Universidad de Desarrollo, Vitacura, Santiago Chile

## Abstract

B7-H4 (VTCN1) is a member of the CD28/B7 family of immune co-inhibitory molecules. The relationship of tumor and stromal B7-H4 protein expression with PD-L1, tumor infiltrating lymphocytes (TILs) and its association with clinico-pathological variables are not well defined. Herein, we explore the expression level of B7-H4 protein in breast cancer and evaluate its association with TILs, levels of PD-L1 expression, and clinico-pathological characteristics in two independent populations. In this study, we used multiplexed automated quantitative immunofluorescence (QIF) to measure the levels of B7-H4 and PD-L1 protein and determined TILs through pathologist assessment of H&E-stained preparations in over a thousand breast cancer cases from two institutions represented in tissue microarray format. Associations between the marker levels, major clinico-pathological variables, and survival were analyzed. We detected B7-H4 protein was highly expressed in both breast cancer and stromal cells. Its expression was independent of breast cancer intrinsic subtypes. PD-L1 expression was higher in triple negative breast cancers. Neither B7-H4 nor PD-L1 were associated with survival in breast cancer. Our study shows there is a mutually exclusive pattern of B7-H4 with both tumor PD-L1 expression and TILs in all breast cancers, independent of breast cancer intrinsic subtype. This exclusive pattern suggests that some breast tumors may preferentially use one B7-related immune evasion mechanism/pathway. This could explain the clinical benefit that is seen only in a fraction of patients with immune checkpoint inhibitors directed exclusively towards PD-L1 in breast cancer.

## Tumor immunity: Immune-inhibitory molecules show mutually exclusive expression patterns

The immune-inhibiting protein B7-H4 is mostly found on breast tumors in which another immune-suppressing protein, PD-L1, and tumor-infiltrating immune cells are both absent. Mehmet Altan from the University of Texas MD Anderson Cancer Center in Houston, USA, and colleagues demonstrated this mutually exclusive pattern of B7-H4 and PD-L1 activity two independent populations—a finding that could explain why drugs designed to boost anti-cancer immunity by blocking PD-1/PD-L1 signaling only work for a fraction of patients with breast cancer. What’s more, the researchers showed that B7-H4 expression levels didn’t track with tumor subtype, unlike PD-L1, which is more commonly elevated in triple-negative breast cancer. The expression patterns highlight the need to develop anti–B7-H4 immunotherapies for those patients who aren’t benefiting from today’s generation of PD-1/PD-L1–targeted checkpoint blockade agents.

## Introduction

T cell co-inhibitory molecules belonging to the B7 family in the tumor microenvironment, such as PD-L1 provide critical inhibitory signals and have been recognized as a major immune inhibitory mechanism in diverse solid tumors.^1–4^ Antibodies that inhibit the PD-1/PD-L1 pathway produce durable clinical responses in various solid malignancies, including breast carcinomas, yet it appears to benefit only a subset of breast tumors.^5–11^

B7-H4 (VTCN1) is also a T-cell co-inhibitory molecule and a member of the B7 family. It has limited expression in normal peripheral tissues, such as lung epithelium, whereas it is expressed at higher levels in several human cancers, including breast carcinomas^12,13^ (Supplementary Table 1). B7-H4 can function as a co-inhibitory factor inhibiting CD4+ and CD8+ T-cell proliferation, cytokine production, and generation of alloreactive cytotoxic T-lymphocytes (CTLs) by arresting the cell cycle.^14,15^ In preclinical models, IL-6 and IL-10 can stimulate B7-H4 expression by monocytes, macrophages, and myeloid dendritic cells.^16–18^ Efforts are ongoing to develop new therapies that target B7-H4.^19^

We hypothesized that B7-H4 expression may inversely correlate with PD-L1 levels in human breast cancers. We also queried whether B7-H4, PD-L1, and tumor infiltrating lymphocyte (TIL) levels, differ among intrinsic subgroups of breast cancer, defined by ER, PR, and HER2 expression. Further, we investigated whether expression of these two molecules differed across cell type (tumor vs. stroma), and whether B7-H4 and/or PD-L1 expression are prognostic in early stage breast cancer.

## Results

### B7-H4 expression in breast cancer

**Fig. 1:**
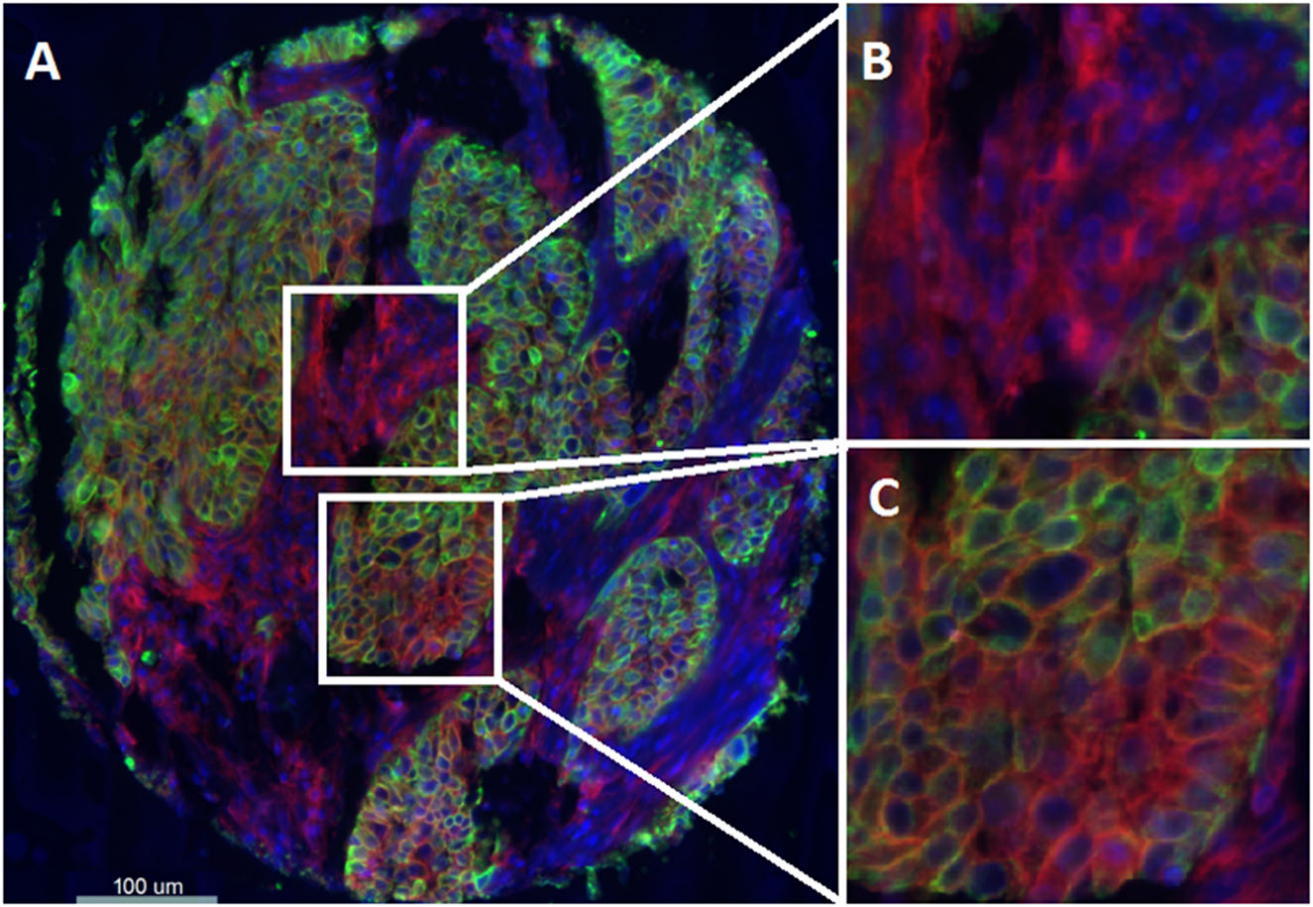
Detection of B7-H4 protein expression by using immunofluorescence (QIF) in breast cancer. **a** Representative fluorescence image showing staining for B7-H4 (red channel), cytokeratin (green channel), and nuclei (4′,6-diamidino-2-phenylindole; DAPI) (blue channel). **b**, **c** Magnified areas of **a**. **b** Target protein, B7-H4 (red channel), is highly expressed within stroma. **c** Target protein, B7-H4 (red channel), and is highly expressed within tumor. Bar = 100 μm

B7-H4 expression was analyzed in 561 and 444 tumors in the Yale and UM cohorts, respectively. B7-H4 protein expression had a predominant cytoplasmic/membranous distribution in tumor and was seen both in tumor and stroma (Fig. 1). The expression in the stroma did not appear to be specific to any specific cell type. Although we measure a continuous score for B7-H4 expression, the visually defined threshold was used to split the population into high/positive vs. low/negative expression.

B7-H4 tumor protein expression in the tumor cell component was high in 257/561 (45.8%) of the Yale cohort patients and 198/444 (44.6%) of the UM cohort patients (Table 1; Supplementary Fig. 1a, b). Stromal B7-H4 protein expression was high in 164/561 (29.2%) and 116/444 (26.1%) in the Yale and UM cohorts, respectively. B7-H4 protein expression of the tumor and stroma was highly correlated in both cohorts (Supplementary Fig. 1c, d). B7-H4 stromal and tumor protein expression was independent of the age, ER/HER2 status, and stage in both the Yale and UM cohorts (Supplementary Table 2).

**Table 1:** Distribution of B7-H4, PD-L1 tumor, and stroma QIF and TILs quantification by H&E in Yale and University of Michigan (UM) cohorts

Variable	Yale	Cohorts	UM	Cohorts
	*N* = 561^a^	%	*N* = 473^a^	%
*B7-H4 tumor*				
Positive	257	45.8	198	44.6
*B7-H4 stroma*				
Positive	164	29.2	116	26.1
*PD-L1 tumor*				
Positive	43	7.7	77	17
*PD-L1 stroma*				
Positive	55	9.8	61	13.6
*PD-L1 any positivity*				
Positive	72	12.8	98	21.8
*TILs*				
0–10	170	41.8	212	50.5
11–20	88	21.6	60	14.3
21–30	75	18.4	52	12.4
31–40	17	4.2	22	5.2
41–50	13	3.2	14	3.3
>50	44	10.8	60	14.3
*TILs*				
Low	363	89.2	360	85.7
High	44	10.8	60	14.3

^a^The actual number of cases for analysis for each marker is different due to loss of tissue or the absence of tumor cells in some spots

### PD-L1 expression in breast cancer

PD-L1 protein expression in the tumor cell component was present in 43/561 (7.7%) and 77/452 (17%) of the Yale and UM cohorts and stromal PD-L1 protein expression was detectable in 55/561 (9.8%) and 61/449 (13.6%) of the Yale and UM cohorts, respectively. Total PD-L1 expression tumor and/or stroma was detected in 12.8% and 21.8% of the Yale and UM cohorts, respectively (Table 1). PD-L1 expression in tumor and in stroma was higher in TNBC and this difference across other breast cancer subtypes was statistically significant in both Yale and UM cohorts (Table 2 and Supplementary Table 3). In combined analysis of the two cohorts, PD-L1 tumor expression was observed in 53/617 (9%) ER-positive cases, compared to 64/341 (19%) ER-negative cases. Likewise, PD-L1 stromal expression was observed in 7% vs. 21% in ER positive vs. negative cases, respectively. Otherwise there was no other clinico-pathological associations found in both of the Yale and UM series for PD-L1 protein expression (Supplementary Table 4).

**Table 2:** Comparison of B7-H4 and PD-L1 protein expression in tumor and stroma for breast cancer intrinsic subtypes (merged data for Yale and UM Cohorts)

Yale and UM cohorts merged^a^	B7-H4 positive		PD-L1 positive	
	Tumor	Stroma	Tumor	Stroma
Triple negative	65/124 (52%)	43/124 (35%)	34/123 (28%)	38/123 (31%)
ER or PR positive/HER2 positive	23/50 (46%)	17/50 (34%)	4/47 (9%)	3/47 (6%)
ER or PR positive/HER2 negative	236/476 (49%)	137/476 (29%)	48/481 (10%)	37/478 (8%)
ER or PR negative/HER2 positive	23/51 (45%)	8/51 (16%)	6/50 (12%)	10/50 (20%)
*p*-value^b^	0.79	0.08	**<0.0001**	**<0.0001**

^a^Triple negative only Yale cohort is excluded from the pooled analysis

^b^Difference in the proportion of positive B7-H4 or PD-L1 between subgroups using chi-square test. *p*-values are considered statistically significant if <0.05 and are indicated in bold

**Fig. 2:**
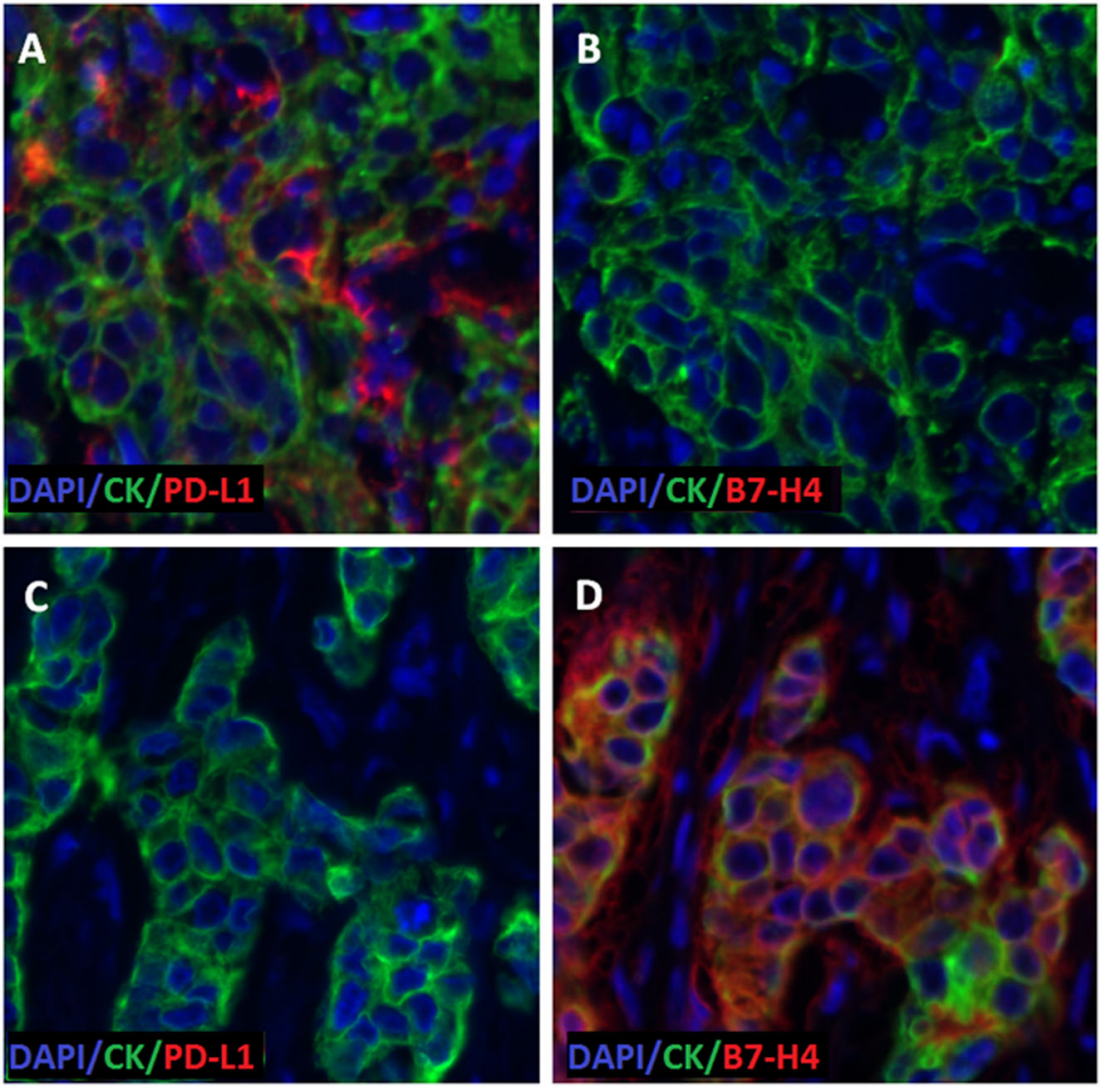
Representative fluorescence image illustrating inverse correlation of tumor PD-L1 and B7-H4 protein expression. Serial sections of two different cases (A, B = case 1; C, D = Case 2). Cytokeratin (green), DAPI (Blue). **a**, **c**: PD-L1 staining (red). **b**, **d**: B7-H4 staining (red), PD-L1 is high in tissue from case 1 in (**a**), and low in tissue from case 2 in (**c**). B7-H4 protein expression is low in tissue from case 1 in (**b**) and high in tissue from case 2 in (**d**)

Co-expression of the two checkpoint proteins in the tumor was quite low in both cohorts (illustration of mutually exclusive expression of PD-L1 (Fig. 2a, c); B7-H4 (Fig. 2b, d)). Co-expression was observed in only 6/561 (1%) cases in the Yale cohorts and 17/424 (4%) cases in the UM cohort, with similar results in the stromal components (Table 3; Supplementary Fig. 2a, b).

**Table 3:** Association of B7-H4, PD-L1 expression by QIF and TILs count in tumor and stromal components of primary breast cancer

Yale cohorts				
	*PDL1*		*N* ^a^	*p*-Value^b^
	Negative	Positive		
*Tumor B7H4*				
Negative	267 (47%)	37 (7%)	304	**<0.0001**
Positive	251 (45%)	6 (1%)	257	
	518	43	561	
*Stromal B7H4*				
Negative	292 (60%)	37 (7%)	329	**0.0018**
Positive	156 (32%)	5 (1%)	161	
	448	42	490	

	*TIL*		*N*	*p*-Value
	Low	High		
*Tumor B7H4*				
Negative	147 (36%)	34 (8%)	181	**<0.0001**
Positive	216 (53%)	10 (3%)	226	
	363	44	407	
*Stromal B7H4*				
Negative	242 (59%)	39 (10%)	281	**0.003**
Positive	121 (30%)	5 (1%)	126	
	363	44	407	
*Tumor PD-L1*				
Negative	359 (88%)	7 (2%)	366	**<0.0001**
Positive	4 (1%)	37 (9%)	41	
	363	44	407	
*Stromal B7H4*				
Negative	331 (81%)	25 (6%)	356	**<0.0001**
Positive	32 (8%)	19 (5%)	51	
	363	44	407	

**UM cohort**				
	*PDL1*		*N* ^a^	*p*-Value^b^
	Negative	Positive		
*Tumor B7H4*				
Negative	175 (41%)	59 (14%)	234	**<0.0001**
Positive	173 (41%)	17 (%4)	190	
	348	76	424	
*Stromal B7H4*				
Negative	265 (63%)	45 (11%)	310	0.82
Positive	95 (23%)	15 (3%)	110	
	360	60	420	

	*TIL*		*N*	*p*-Value
	Low	High		
*Tumor B7H4*				
Negative	174 (41%)	52 (12%)	226	**<0.0001**
Positive	188 (45%)	7 (2%)	195	
	362	59	421	
*Stromal B7H4*				
Negative	249 (59%)	57 (13%)	306	**<0.0001**
Positive	113 (27%)	3 (1%)	116	
	362	60	422	
*Tumor PD-L1*				
Negative	323 (78%)	21 (5%)	344	**<0.0001**
Positive	34 (8%)	38 (9%)	72	
	357	59	416	
*Stromal B7H4*				
Negative	317 (77%)	35 (8%)	352	**<0.0001**
Positive	37 (9%)	24 (6%)	61	
	354	59	413	

^a^Only cases which had staining for both markers were included to analysis

^b^QIF signals between groups presented in frequency tables evaluated by Chi-Square test. Two sided *p*-values are considered statistically significant if <0.05 and are indicated in bold

### Association of B7-H4 with PD-L1 and TIL Infiltration

More than 10% T-cell infiltration was observed in 58.2% and 49.5% of the Yale and UM cohorts, respectively, 10.8% and 14.3% of the Yale and UM cohorts were considered to have elevated TIL levels using 50% as a cut off (Table 1). PD-L1 and B7-H4 were inversely correlated. Furthermore, TILs were weakly, positively correlated with PD-L1 but weakly, inversely correlated with B7-H4 expression in the UM cohort and weakly positively correlated with PD-L1 and B7-H4 expression in the Yale cohorts. Both cohorts, however, had a high incidence of low staining for both markers and low levels of TILs (Supplementary Table 5, Supplementary Fig. 3). Nonetheless, B7-H4 was positive and TILs were high in only 10/407 (2%); and 7/421 (2%) whereas in cases with high TILs, PD-L1 over expression was (37/44) 84% and (38/59) 64% of the Yale and UM cohorts, respectively (Table 3).

### Association of B7-H4 and PD-L1 and survival

**Fig. 3:**
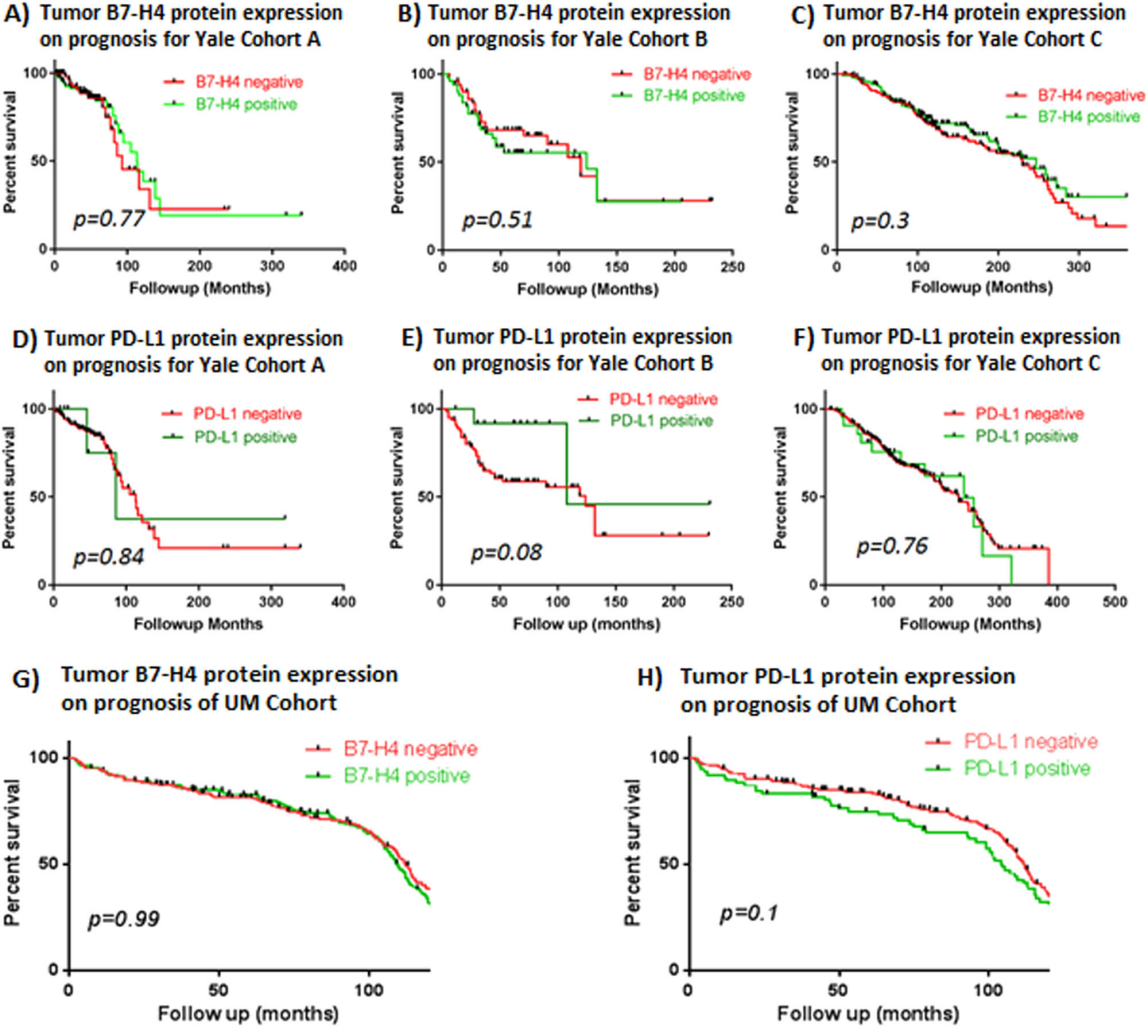
Survival curves according to B7-H4 or PD-L1 expression in Yale and UM cohorts. **a**–**f** Three Yale cohorts [(Cohort C = TNBC cohort)); **a**–**c**: B7-H4; D-F: PD-L1], **g**, **h** UM cohort (**g**: B7-H4; **h**: PD-L1). Red lines: negative; green lines: positive protein expression for given marker

Neither B7-H4 nor PD-L1 expression in either tumor or stroma was associated with survival outcome in either cohort (Fig. 3). In both cohorts, overall survival (OS) was nearly identical for those with or without elevated B7-H4 expression. Although there was a trend towards worse OS in one of the Yale cohorts and in the UM cohort, neither of these met statistical significance.

## Discussion

In this study, we have observed that the immune checkpoint B7-H4 is expressed in the epithelial tumor component of ~50% of breast tumors, and in the stromal element in ~25–30% of cases. Furthermore, it was unusual to have both B7-H4 and PD-L1 expressed in the same tumor, suggesting nearly total mutual exclusion. PD-L1 was weakly correlated with TILs in both cohorts, whereas B7-H4 was weakly positively correlated with TILs in one cohort and weakly negatively correlated in the other.

Taken together, these results suggest that breast cancers may employ one or the other, but rarely simultaneous, immune checkpoint pathways to evade immunity. Moreover, B7-H4 expression was not associated with any of the particular intrinsic subtypes of breast cancer, defined by expression of ER or HER2. In contrast, as previously reported, PD-L1 was more commonly expressed in TNBC cases.^20,21^

These data may have clinical implications. Immune checkpoint inhibitors have been found to have striking activity in a variety of solid malignancies,^5–8^ including breast cancer, however, they are effective in fraction of patients.^9,22–24^ Our data suggest that cases lacking PD-L1 might escape immune surveillance by virtue of upregulation of B7-H4 or other non-redundant alternative targets.

Most clinical trials of immune checkpoint inhibition have been focused on TNBC, since PD-L1 is more likely, although not exclusively, expressed in this sub-type, and there are few other treatment options except chemotherapy. Our data demonstrate that B7-H4 is not only inversely related to PD-L1 expression but also its expression appears to be independent of breast cancer intrinsic subtype.

In our study, neither B7-H4 nor PD-L1 expression was associated with patient outcome, although we detected a statistically insignificant trend towards worse survival for over-expression of the latter. However, our datasets were serially collected retrospective studies, with mixes of intrinsic subtypes and a variety of treatments.^25^ It is possible that either or both of the immune checkpoints might be prognostic in subgroups of patients according to hormone receptor or HER2 expression, or according to specific treatments. However, our power to conduct such subgroup analyses was limited. Nonetheless, we speculate that these molecules may serve as general mechanisms of tolerance to the immune system employed by epithelial tissue, and identification of mechanism of tolerance and breaking tolerance with checkpoint specific or with use of combined immune checkpoint inhibitors may result in reduction in tumor burden and patient benefit.

The presence of increased TILs is associated with increased PD-L1 levels in diverse tumors suggesting that these factors are biologically linked.^26–28^ It has been postulated that B7-H4 overexpression in immune cells may impair the immune response against tumors by decreasing the TILs via various mechanisms. A study revealed that T cells isolated from cervical cancer patients co-cultured with recombinant B7-H4 for 48 h, which resulted activated T cell arrest at the G1/G2 phase,^29^ another experiment with co-cultured cytotoxic lymphocytes with Lewis lung carcinoma cell line showed that membrane bound B7-H4-induced CTL apoptosis which was partially reversed when B7-H4 blocked with a neutralizing monoclonal antibody.^30^ Decreased TILs in the tumor microenviroment decreases production of pro-inflammatory cytokines such as IFN-γ. This phenomenon might alter the immune balance towards tumor-associated FOXP3+ T reg cells, which can trigger APCs including macrophages to produce inhibitory cytokines, such as IL-6 and IL-10.^29,31–33^

Number of preclinical studies support the hypothesis of B7-H4 generating an inhibitory tumor microenviroment, for example, in one study it has been shown that the quantity of B7-H4 on the surface of pancreatic islets cells positively correlates with their resistance to T cell attack in murine models of type 1 diabetes.^34^ In another study when 4T1 metastatic breast cancer cells were transferred into both wildtype and B7-H4^−/−^ mice. In B7-H4^−/−^ mice, the percentage and overall number of CD4+ Foxp3+ Tregs was reduced and a significantly higher ratio of effector CD4+ and CD8+ T cells was observed.^35^ Indeed, our results, showing an inverse relationship of B7-H4 and TILs are consistent with this hypothesis. In this regard, our group recently reported similar findings in non-small cell lung cancer, further supporting the biologic balance of PD-L1 and B7-H4 to control immune response across different tumor types.^36^ A scenario can be envisioned in which a B7-H4 drug is only effective in combination with a PD-L1 inhibitor. B7-H4 inhibition might trigger lymphocyte infiltration and activate an immune response that could in turn be complemented by a PD-1 axis checkpoint inhibitor. However, with the existing data, we can only state that the tumors that express B7-H4 appear to be a different subset than those that are more likely to respond to current immune therapies, as indicated by TILs or PD-L1 expression.

The lack of concordance of these markers, especially PD-L1 in the tumor and stroma compartments (Supplementary Fig. 5) could be explained by the expression of PD-L1 in macrophages and morphologically fibroblast-like cells in addition to TILs in breast cancer stroma, as previously described.^37^ Future studies focused on phenotype of these cells would provide further evidence.

This study has number of limitations. The protein expression and TILs assessments were performed in archival tissue without serial biopsies, thus, we cannot assess the temporal dynamics of protein expression and lymphocytic infiltration. Further, the work was entirely done on TMAs. The use of TMAs may not provide the same information as if the expression patterns had been performed on whole sections. It is reassuring that the TMA data were comparable in cohorts from two different institutions. Also defining the cut points are difficult to determine due to the nature of the continuous data generated in these studies. In this work, visual cut off is selected after reviewing many images and after the specific staining pattern, which has been perimembranous/cytoplasmic for the B7-H4, subjectively visualized. Once a cut off is established the same cut off is used for all cohorts to be consistent throughout the study.

In summary, we report that a second immune checkpoint protein, B7-H4, is commonly expressed in breast cancer, that its expression is independent of intrinsic subtypes, and that it is inversely related to PD-L1 and TILs. Although not related to prognosis in standardly treated patients with early stage disease, we speculate that these findings could have implications for further development and studies of immune checkpoint inhibitors in this disease.

## Methods

### Patients, cohorts, and tissue microarrays (TMAs)

Samples from four retrospective collections of breast cancer, three from Yale University (cohorts A–C) and one from University of Michigan (UM) represented in TMAs were used. The major clinico-pathological characteristics of the cohorts are presented in Supplementary Table 6. Two of the Yale University cohorts (cohorts A and B) have been previously described^38,39^ and a third cohort (cohort C) consists of tissues collected from patients with stages I–III triple negative breast cancer (TNBC). The fourth, cohort D consists of a set of 473 breast cases treated at the University of Michigan Comprehensive Cancer Center (UMCCC).

Yale University cohorts were retrospectively derived from stages I–III breast cancer collections of patients who underwent surgical tumor resection and were followed at Yale University from 1976 to 2010.

Cases in cohort D were derived from all patients who had definitive surgery for invasive breast cancer at UMCCC between 2004 and 2005, and for whom breast cancer tissue blocks were therefore available, and who underwent subsequent therapy and follow-up at UMCCC.

Tissue and associated clinico-pathological information was used after approval from the Yale Human Investigation Committee (protocol #9505008219) or from the University of Michigan Institutional Review Board (IRBMED#2001-0788; HUM00042180). Both review boards provided waiver of consent, since these were otherwise discarded tissues collected during routine medical care.

Two different built TMA blocks from each cohort using 0.6 mm tissue cores were included. These blocks were constructed with the same TMA map with cores obtained by nonadjacent sampling of the same tumor blocks to examine tumor heterogeneity.

### Antibody validation-cell line transfection and assessment for B7-H4 and PD-L1

Open-access databases (Expression Atlas European Bioinformatics Institute EMBL-EBI, https://www.ebi.ac.uk/gxa/home and Cell Line Atlas-The Human Protein Atlas, www.proteinatlas.org/cell) were reviewed for cell line mRNA and protein expression of B7-H4 (VTCN1). The cultured human MCF7 breast cancer cell line (American Type Culture Collection) was selected for negative control due to low mRNA expression.

MCF7 cells were seeded in 24-well plates in duplicates in Gibco™ RPMI Medium (ThermoFisher Scientific, USA), supplemented with 10% FBS, Penicillin–Streptomycin (10,000 U/ml) and incubated at 37 °C, 5% CO_2_. When they reached 70% confluency, they were transferred to Optimem-Low Serum Medium (ThermoFisher Scientific, USA), transfected with 500 ng of B7-H4 plasmid (provided by Cell Signaling Technologies (CST), MA) with different Lipofectamine 2000 concentrations ranging from 2 to 5 µl (ThermoFisher, USA), following manufacturer’s instructions. Empty vector and untreated cells (no plasmid, no lipofectamine) were included as negative controls. After 72 h incubation, cells were collected and replated in eight-chamber polystyrene vessel tissue culture-treated glass slides (Falcon, Cat. no. 354108) in duplicates. Glass slides were washed twice in PBS (Life Technologies) and fixed in 4% paraformaldehyde for 10 min. Then, following double wash in PBS they were permeabilized in 0.2% Triton x100-PBS for 3 min.

After a double wash in PBS followed by blocking in 2% BSA-PBS for 1 h at room temperature, primary antibodies against cytokeratin (monoclonal mouse antihuman pan-cytokeratin (clone AE1/AE3, M3515; Dako Corp., Carpinteria, CA, USA)) B7-H4 (monoclonal rabbit antibody, Cell Signaling Technologies, clone D1M8I) at a working concentration of 1.305 μl/ml were added overnight at 4 °C, in a light protected chamber. After two washes of 1% tween-PBS and one in PBS 5 min each, secondary antibodies were added using GAM/Alexa Fluor 546-conjugated goat anti-mouse secondary antibody (Molecular Probes, Eugene, OR, USA) diluted 1:100 in rabbit EnVision reagent (Dako) for 1 h at room temperature. After PBS-tween/PBS wash Cyanine 5 (Cy5) directly conjugated to tyramide (Perkin-Elmer, Waltham, MA, USA) at 1:50 dilution in amplification buffer was added for 10 min for target antibody detection. Finally, after a last PBS-tween/PBS wash, mounting was performed with Prolong Gold-DAPI (Life Technologies). Quantitative immunofluorescence (QIF) microscopy used for assessment of protein expression of both positive and negative controls in MCF7 for B7-H4 transfection (Supplementary Fig. 4).

For PD-L1 QIF we used a validated antibody (clone SP142 Spring BioScience) as previously reported by our group, at a working concentration of 0.1 μl/ml.^40,41^

### Quantitative immunofluorescence

We measured the levels of B7-H4 (clone D1M8I, CST), PD-L1 (clone SP142, Spring Bioscience) combined with pancytokeratin to determine the tumor and stromal compartment by QIF in TMA slides containing the cohort cases. Serial section slides were used for analyzing the staining and protein expression patterns for the two markers. TMA slides were baked overnight at 60 °C and then soaked in xylene twice for 20 min each. Slides were rehydrated in two 1 min washes in 100% ethanol followed by one wash in 70% ethanol and finally rinsed in streaming tap water for 5 min. Antigen retrieval was performed in sodium citrate buffer, pH 6 for B7-H4 antibody and with EDTA buffer, pH 8 for PD-L1 antibody in the PT module-LabVision for 20 min at 97 °C in a pressure-boiling container. Blocking was performed with 0.3% bovine serum albumin in 0.05% tween solution for 30 min after antigen retrieval. Each of the B7 family antibodies (B7-H4 and PD-L1) was combined with 1:100 pan-cytokeratin antibody (Dako) in 0.3% BSA in TBST and incubated overnight at 4 °C. Primary antibodies were followed by incubation with Alexa 546-conjugated goat anti-mouse or anti-rabbit secondary antibody (Molecular Probes, Eugene, OR, USA) diluted 1:100 in rabbit or mouse EnVision reagent (Dako) for 1 h. Signal was amplified with Cyanine 5 (Cy5) directly conjugated to tyramide (Perkin-Elmer, Waltham, MA, USA) at 1:50 dilution. ProLong mounting medium (ProLong Gold; Molecular Probes) with 4,6-diamidino-2-phenylindole (DAPI) was used to stain nuclei.

The QIF measurements were performed using the AQUA^®^ method of QIF (Genoptix Medical Laboratory) as previously described.^42^ The QIF score of B7-H4 antibody in the tumor and in the stroma was calculated by dividing the target B7-H4 pixel intensity in the area of the tumor and stroma compartment defined by the cytokeratin positivity in tumor cells.

In order to better represent tumor heterogeneity in the quantitative analysis of B7-H4 and PD-L1 protein expression, we performed QIF staining in two different TMA builds. To address run-to-run variability, slides were stained and analyzed on the same day using the same protocol. Serial cut index slides were used for quality control and regression coefficients (*R*^2^) between independent runs for these index slides were found to be high (>0.9). Two TMA histospots were evaluated for each case and the average score was determined. Cases which had <2% tumor tissue, extensive necrosis or non-invasive tumor tissue in a histospot were excluded from the analysis.

### TILs assessment

Stromal TILs were assessed in H&E-stained TMA sections by two experienced Yale pathologists (V.P., D.C.H.) and expressed as the percentage of total cells using 10% increments as suggested by International TILs Working Group.^43^ Sections with 50% or greater TIL infiltrate were denoted as lymphocyte predominant breast cancer.^43^

### ER, PR, and HER2 determination

ER, PR, and HER2 were collected from the clinical charts at both institutions. All of the specimens from both institutions were collected from cases that preceded the 2010 ASCO/CAP guidelines for ER/PR.^44^ Any case with more than 10% of tumor cells showing reactivity in the nucleus was called positive. For HER2, the conventional scoring system was used including a four-point subjective scale (0–3). HER2 IHC staining of 3+ (uniform, intense membrane staining of >30% of invasive tumor cells) and HER2 IHC staining of +2 (were considered equivocal) that were re-assayed for gene amplification using FISH as suggested in ASCO/CAP 2007 guidelines.^45^

### Statistical analysis

All results are presented using REMARK criteria.^46^ The three breast cancer cohorts from Yale University were pooled for the analysis. The fourth cohort from UM was analyzed separately. A visual cut point was determined for QIF positivity and same cut off was used for all cohorts. Pearson’s correlation coefficient (*R*) was used to assess the reproducibility of the assay between near-serial sections of the index array. Differences between QIF signals between groups were analyzed using Fisher’s exact test. Two-sided *p*-value < 0.05 was considered statistically significant. Linear regression was used to determine the association between continuous scores after a natural log transformation of the data to satisfy linearity assumptions and the coefficient of determination (*R*^2^) was calculated. Chi-square tests were performed to compare characteristics between groups. Spearman’s correlation was used to assess the correlation between TILs and PD-L1 and B7-H4 expressions.

Survival analysis of continuous marker scores for the Yale combined cohorts was performed using the X-tile software (Yale University, New Haven, CT, USA) for disease-specific survival differences.^47^ This analysis exported to GraphPad Prism 7.01 software for Kaplan–Meier OS curve presentations. Statistical analysis system (SAS) v 9.4 was used for analysis of the Michigan cohort.

## Data Availability

The data analyzed during this study are described in the following metadata record: 10.6084/m9.figshare.c.4304642.^48^ Expression data are available from the EMBL-EBI Expression Protein Atlas and Human Protein Atlas as outlined in the following metadata record: 10.6084/m9.figshare.7352912. Quantitative immunofluorescence image data are available on request as outlined in the following metadata record: 10.6084/m9.figshare.7352834. Additional analyzed data files are available in the Supplementary Information of this article.

## Supplementary information

Supplemental Material

## Electronic supplementary material

**Supplementary information** accompanies the paper on the *npj Breast Cancer* website (10.1038/s41523-018-0095-1).
