# Supplementary material for: Association of B7-H4, PD-L1, and tumor infiltrating lymphocytes with outcomes in breast cancer
Source: NPJ Breast Cancer. 2018 Dec 10;4:40. doi: 10.1038/s41523-018-0095-1 (PMC6288133; doi:10.1038/s41523-018-0095-1)
Supplement: Supplementary file 1 — Supplemental Material [file 41523_2018_95_MOESM1_ESM.docx]

**Supplemental figures and tables:**

**Supp. Table 1.** Examples of B7-H4 Protein expression in different tumor types in literature

**Supp. Table 2.** Association of clinic-pathologic features of tumor and stroma B7-H4 protein expression in Yale and UM Cohorts

**Supp. Table 3.** Association of clinic-pathologic features of tumor and stroma PD-L1 protein expression in Yale and UM Cohorts

**Supp. Table 4.** Comparison of B7-H4 and PD-L1 protein expression in tumor and stroma for breast cancer intrinsic subtypes in Yale and UM Cohorts

**Supp.** Table 5 A and B: Spearman Correlation Matrix for Yale Cohorts and University of Michigan (UM) Cohorts

**Supp. Table 6.** Clinico-pathological features of the Yale Cohorts and University of Michigan (UM) Cohorts

**Supp.** **Figure 1.** Expression of B7-H4 in tumor and stromal components of primary breast cancers as determined by QIF

**Supp Figure 2.** Concordance of B7-H4 and PD-L1 scatter plots for Yale (A) and UM (B) cohorts.

**Suppl Figure 3.** Scatter plots for TILs, PD-L1 and B7-H4 in Yale and UM Cohorts

**Supp. Figure 4.** B7-H4 antibody validation

**Supp. Table 1:** Examples of B7-H4 Protein expression in different tumor types studies with different antibodies in literature

| **First author (ref)** | **Cancer** | **Case number** | **Antibody used** | **Scoring method** | **Cut off value** | **Protein exp rate** | **Major clinic histologic findings/**  **outcome measures** |
| --- | --- | --- | --- | --- | --- | --- | --- |
| Tringer et al.^1^ | Breast cancer | 173 | A57.1 monoclonal Antibody | Semi quantitative IHC | 0-10% positive cells, >10-50% positive cells, >50-80% positive cells, and >80-100% positive cells | B7-H4 expression was detected in invasive breast cancers, including 147 of 155 (94.8%) cases of invasive ductal carcinoma and 18 of 18 (100%) cases of invasive lobular carcinoma | B7-H4 circumferential membranous and cytoplasmic expression was observed in >95% of invasive breast cancer cases and was also detected in most nodal metastases |
| Sun et al.^2^ | NSCLC | 70 | Anti B7-H4 (Nordic Biosite AB) | Semi quantitative IHC | Negative group (focal expression < 10%) and a positive group (focal expression in 10–100%) | 30/70 (42.9%) B7-H4 focal expression < 10% reported | There was no statistical differences between histology, age, sex, smoking hx or differentiation |
| Quandt et al. ^3^ | Melanoma | 29 | αB7-H4, clone H74, eBioscience | Semi quantitative IHC | Scores of 0 to 5 were given distinguishing low (scores 0–3) and high (scores 4 and 5) | 97% 28 of 29 samples reported to have high score (4-5/5) | Tumor-associated B7-H4 had an influence on T cell cytokine production. A marked inhibition of IFNγ production of these different T cells on coculture with B7-H4 overexpressing melanoma cells reported |
| Liang et al.^4^ | Ovarian Serous carcinoma | 306 | Anti-B7-H4, clone D1M8I, Cell Signaling, Danvers, MA | Semi quantitative IHC | 4-score grading system (Score 0, no staining/negative; Score 1, apical pattern; Score 2, mixed apical and circumferential membranous staining with circumferential membranous staining in <10% of tumor cells; and Score 3, circumferential membranous staining in ≥10% of tumor cells) | 91% (267/293) of the high-grade ovarian serous carcinomas and 69% (9/13) of the low-grade ovarian serous carcinomas expressed B7-H4 | No significant association between B7-H4 immunohistochemical score and OS or DFS was found |
| Huang et al. ^5^ | Breast Cancer | 147 | ab110221; abcam | Semi quantitative IHC | 4-score grading system (no staining = 0; weak staining = 1; moderate staining = 2; strong staining = 3) | 89/147 samples had strong positive staining | High B7-H4 expression was correlating with lower overall survival |

**NSCLC: Non-small cell lung cancer, OS: Overall survival, DFS: Disease free survival**

**Supp. Table 2.** Association of clinico-pathologic features of tumor and stroma B7-H4 protein expression in Yale and UM Cohorts

|  | **Yale** | **Cohorts** |  |  |  | **UM** | **Cohort** |  |
| --- | --- | --- | --- | --- | --- | --- | --- | --- |
|  |  | **Tumor B7H4** |  |  |  |  | **Tumor B7H4** |  |
| **Variable** | **N*** | **Positive (%)** | **p value**** |  | **Variable** | **N*** | **Positive (%)** | **p value**** |
| **Age** |  |  |  |  | **Age** |  |  |  |
| **<50** | 197 | 110 (55.8%) | 0.72 |  | **<50** | 155 | 61 (39.3%) | 0.13 |
| **≥50** | 352 | 190 (53.9%) |  |  | **≥50** | 289 | 137 (47.4%) |  |
| **ER** |  |  |  |  | **ER** |  |  |  |
| **Negative** | 216 | 119 (55%) | 0.78 |  | **Negative** | 126 | 52 (41.2%) | 0.46 |
| **Positive** | 293 | 166 (56.6%) |  |  | **Positive** | 315 | 144 (45.7%) |  |
| **PR** |  |  |  |  | **PR** |  |  |  |
| **Negative** | 234 | 128 (54.7%) | 0.59 |  | **Negative** | 202 | 85 (42%) | 0.45 |
| **Positive** | 275 | 157 (57%) |  |  | **Positive** | 234 | 108 (46.1%) |  |
| **Her 2** |  |  |  |  | **Her 2** |  |  |  |
| **Negative** | 325 | 182 (56%) | 1 |  | **Negative** | 370 | 166 (44.8%) | 0.83 |
| **Positive** | 29 | 16 (55.1%) |  |  | **Positive** | 59 | 25 (42.3%) |  |
| **Stage** |  |  |  |  | **Stage** |  |  |  |
| **I and II** | 448 | 249 (55.5%) | 0.27 |  | **I and II** | 406 | 185 (45.5%) | 0.17 |
| **III and IV** | 46 | 30 (65.2%) |  |  | **III and IV** | 37 | 12 (32.4%) |  |
|  |  |  |  |  |  |  |  |  |
|  | **Yale** | **Cohorts** |  |  |  | **UM** | **Cohort** |  |
|  |  | **Stroma B7H4** |  |  |  |  | **Stroma B7H4** |  |
| **Variable** | **N*** | **Positive (%)** | **p value**** |  | **Variable** | **N*** | **Positive (%)** | **p value**** |
| **Age** |  |  |  |  | **Age** |  |  |  |
| **<50** | 197 | 57 (28.9%) | 1 |  | **<50** | 154 | 36 (23.3%) | 0.4 |
| **≥50** | 352 | 102 (28.9%) |  |  | **≥50** | 290 | 80 (27.5%) |  |
| **ER** |  |  |  |  | **ER** |  |  |  |
| **Negative** | 216 | 60 (27.7%) | 0.43 |  | **Negative** | 125 | 29 (23.2%) | 0.42 |
| **Positive** | 293 | 92 (31.3%) |  |  | **Positive** | 316 | 87 (27.5%) |  |
| **PR** |  |  |  |  | **PR** |  |  |  |
| **Negative** | 220 | 51 (23.1%) | 0.04 |  | **Negative** | 201 | 46 (22.8%) | 0.13 |
| **Positive** | 289 | 101 (34.9%) |  |  | **Positive** | 235 | 70 (29.7%) |  |
| **Her 2** |  |  |  |  | **Her 2** |  |  |  |
| **Negative** | 325 | 98 (30.1%) | 0.4 |  | **Negative** | 369 | 102 (27.6%) | 0.31 |
| **Positive** | 29 | 11 (37.9% |  |  | **Positive** | 59 | 12 (20.3%) |  |
| **Stage** |  |  |  |  | **Stage** |  |  |  |
| **I and II** | 448 | 135 (30.1%) | 0.5 |  | **I and II** | 406 | 111 (27.3%) | 0.1 |
| **III and IV** | 46 | 16 (34.7%) |  |  | **III and IV** | 37 | 5 (13.5%) |  |

*Analysis (chi-square tests) includes 561 tumors in Yale and 444 tumors in UM cohort, clinico-pathologic information is missing in some cases. ** Two sided p-values are considered statistically significant if <0.05 and highlighted in red.

**Supp. Table 3.**  Comparison of B7-H4 and PD-L1 protein expression in tumor and stroma for breast cancer intrinsic subtypes in Yale and UM Cohorts.

| **Yale Cohorts*** | **B7-H4 Positive** | | **PD-L1 Positive** | |  |
| --- | --- | --- | --- | --- | --- |
|  | **Tumor** | **Stroma** | **Tumor** | **Stroma** |  |
| Triple Negative | 29/42 | 19/42 | 8/41 | 14/41 |  |
| ER or PR positive / HER2 positive | 7/18 | 6/18 | 1/16 | 0/16 |  |
| ER or PR positive / HER2 negative | 106/189 | 59/189 | 6/184 | 10/184 |  |
| ER or PR negative / HER2 positive | 14/24 | 7/24 | 1/22 | 4/22 |  |
| *p*-value** | 0.17 | 0.36 | 0.003 | ‹0.0001 |  |
|  |  |  |  |  |  |
| **UM Cohorts** | **B7-H4 Positive** | | **PD-L1 Positive** | | |
|  | **Tumor** | **Stroma** | **Tumor** | **Stroma** | |
| Triple Negative | 36/82 | 24/82 | 26/82 | 24/82 | |
| ER or PR positive / HER2 positive | 16/32 | 11/32 | 3/31 | 3/31 | |
| ER or PR positive / HER2 negative | 130/287 | 78/287 | 42/297 | 27/294 | |
| ER or PR negative / HER2 positive | 9/27 | 1/27 | 5/28 | 6/28 | |
| *p*-value** | 0.61 | 0.035 | 0.002 | ‹0.0001 | |

*Triple negative only Yale cohort is excluded from the pooled analysis. ** Difference in the proportion of positive B7H4 or PDL1 between subgroups from chi-square tests

**Supp. Table 4.** Association of clinic-pathologic features of tumor and stroma PD-L1 protein expression in UM and Yale Cohorts

|  | **Yale** | **Cohorts** |  |  |  | **UM** | **Cohort** |  |
| --- | --- | --- | --- | --- | --- | --- | --- | --- |
|  |  | **Tumor PD-L1** |  |  |  |  | **Tumor PD-L1** |  |
| **Variable** | **N*** | **Positive (%)** | **p value**** |  | **Variable** | **N*** | **Positive (%)** | **p value**** |
| **Age** |  |  |  |  | **Age** |  |  |  |
| **<50** | 197 | 26 (13.1%) | 0.0004 |  | **<50** | 159 | 25 (15.7%) | 0.68 |
| **≥50** | 352 | 16 (4.5%) |  |  | **≥50** | 293 | 52 (17.7%) |  |
| **ER** |  |  |  |  | **ER** |  |  |  |
| **Negative** | 216 | 32 (14.8%) | 0.0001 |  | **Negative** | 125 | 32 (25.6%) | 0.005 |
| **Positive** | 293 | 8 (2.7%) |  |  | **Positive** | 324 | 45 (13.8%) |  |
| **PR** |  |  |  |  | **PR** |  |  |  |
| **Negative** | 234 | 11 (4.7%) | 0.02 |  | **Negative** | 206 | 41 (19.9%) | 0.22 |
| **Positive** | 275 | 29 (10.5%) |  |  | **Positive** | 239 | 36 (15%) |  |
| **Her 2** |  |  |  |  | **Her 2** |  |  |  |
| **Negative** | 325 | 29 (8.9%) | 0.49 |  | **Negative** | 379 | 68 (17.9%) | 0.52 |
| **Positive** | 29 | 1 (3.4%) |  |  | **Positive** | 59 | 8 (13.5%) |  |
| **Stage** |  |  |  |  | **Stage** |  |  |  |
| **I and II** | 448 | 35 (7.8%) | 0.77 |  | **0, I and II** | 416 | 75 (18%) | 0.01 |
| **III and IV** | 46 | 4 (8.6%) |  |  | **III and IV** | 35 | 2 (5.7%) |  |
|  |  |  |  |  |  |  |  |  |
|  | **Yale** | **Cohorts** |  |  |  | **UM** | **Cohort** |  |
|  |  | **Stroma PD-L1** |  |  |  |  | **Stroma PD-L1** |  |
| **Variable** | **N*** | **Positive (%)** | **p value**** |  | **Variable** | **N** | **Positive (%)** | **p value** |
| **Age** |  |  |  |  | **Age** |  |  |  |
| **<50** | 197 | 27 (13.7%) | 0.023 |  | **<50** | 158 | 22 (13.9%) | 0.99 |
| **≥50** | 352 | 26 (7.3%) |  |  | **≥50** | 291 | 39 (13.4%) |  |
| **ER** |  |  |  |  | **ER** |  |  |  |
| **Negative** | 216 | 40 (18.5%) | <0.0001 |  | **Negative** | 125 | 31 (24.8%) | <0.0001 |
| **Positive** | 293 | 14 (4.7%) |  |  | **Positive** | 321 | 30 (9.3%) |  |
| **PR** |  |  |  |  | **PR** |  |  |  |
| **Negative** | 216 | 26 (12%) | 0.38 |  | **Negative** | 205 | 36 (17.5%) | 0.046 |
| **Positive** | 293 | 28 (9.5%) |  |  | **Positive** | 237 | 25 (10.5%) |  |
| **Her 2** |  |  |  |  | **Her 2** |  |  |  |
| **Negative** | 325 | 37 (11.3%) | 0.76 |  | **Negative** | 376 | 51 (13.5%) | 0.88 |
| **Positive** | 29 | 4 (13.7%) |  |  | **Positive** | 59 | 9 (15.2%) |  |
| **Stage** |  |  |  |  | **Stage** |  |  |  |
| **I and II** | 448 | 48 (10.7%) | 0.61 |  | **0, I and II** | 413 | 60 (14.5%) | 0.09 |
| **III and IV** | 46 | 6 (13%) |  |  | **III and IV** | 35 | 1 (2.8%) |  |

*Analysis (chi-square tests) includes 561 tumors in Yale and 444 tumors in UM cohort, clinico-pathologic information is missing in some cases. ** Two sided p-values are considered statistically significant if <0.05 and highlighted in red

**Supp. Table 5: Correlations for Yale and UM Cohorts**

**5. A. Spearman Correlation Matrix for Yale Cohorts**

|  | PDL1 Stroma | PDL1 Tumor | B7H4 Stroma | B7H4 Tumor |
| --- | --- | --- | --- | --- |
| Tils | 0.14 | 0.04 | 0.16 | 0.13 |
| PDL1 Stroma | - | 0.72 | -0.16 | -0.30 |
| PDL1 Tumor | - | - | -0.49 | -0.49 |
| B7H4 Stroma | - | - | - | 0.83 |

**5.B. Spearman Correlation Matrix for UM Cohorts**

|  | PDL1 Stroma | PDL1 Tumor | B7H4 Stroma | B7H4 Tumor |
| --- | --- | --- | --- | --- |
| Tils | 0.16 | 0.46 | -0.19 | -0.19 |
| PDL1 Stroma | - | 0.47 | 0.28 | 0.18 |
| PDL1 Tumor | - | - | -0.10 | -0.12 |
| B7H4 Stroma | - | - | - | 0.90 |

*Correlations between TILs and PDL1 and B7H4 is weaker for Yale cohort than UM. For Yale, there is a weak, positive correlation between TILs and the stroma and tumor expressions, separately. For UM, there is a weak to moderate positive correlation between TILs and PDL1 and a weak negative correlation between TILs and B7H4.

**Supp. Table 6 :** Clinico-pathologic characteristics of Yale and University of Michigan (UM) Cohorts

|  | **Yale** | **Cohorts** | **UM** | **Cohort** |
| --- | --- | --- | --- | --- |
| **Characteristic** | N=654 | % | N=473 | % |
| *Age* |  |  |  |  |
| <50 | 227 | 34.7% | 166 | 35.1% |
| ≥50 | 412 | 63% | 307 | 64.9% |
| Unknown | 15 | 2.3% |  |  |
| *ER* |  |  |  |  |
| Negative | 264 | 40.4% | 132 | 27.9% |
| Positive | 334 | 51% | 338 | 71.5% |
| Unknown | 56 | 8.6% | 3 | 0.6% |
| *PR* |  |  |  |  |
| Negative | 304 | 46.5% | 216 | 45.7% |
| Positive | 290 | 44.3% | 249 | 52.6% |
| Unknown | 60 | 9.2% | 8 | 1.7% |
| *HER2* |  |  |  |  |
| Negative | 392 | 59.9% | 394 | 83.3% |
| Positive | 38 | 5.8% | 63 | 13.3% |
| Unknown | 224 | 34.3% | 16 | 3.4% |
| *Stage* |  |  |  |  |
| 1 | 297 | 45.4% | 305 | 64.5% |
| 2 | 226 | 34.5% | 130 | 27.5% |
| 3 | 56 | 8.6% | 29 | 6.1% |
| 4 |  |  | 8 | 1.7% |
| Unknown | 75 | 11.5% | 1 | 0.2% |

**Supp. Figure 1.**
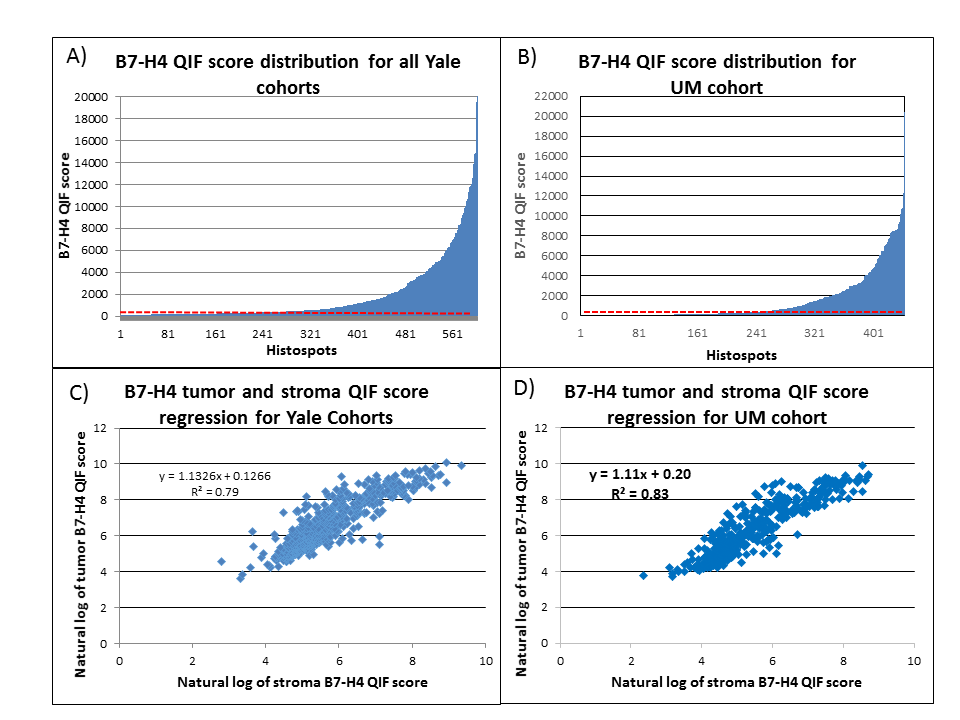


Expression of B7-H4 in tumor and stromal components of primary breast cancers as determined by QIF. A, B: Tumor expression in Yale (A) and UM (B) cohorts. The dashed red line indicates the signal detection threshold determined by visual cut off for B7-H4 protein. C, D: Linear correlation coefficient [R2] for log data for B7-H4 tumor and stroma QIF scores, for Yale (C) and UM (D) cohorts.

**Supp Figure 2.**

**
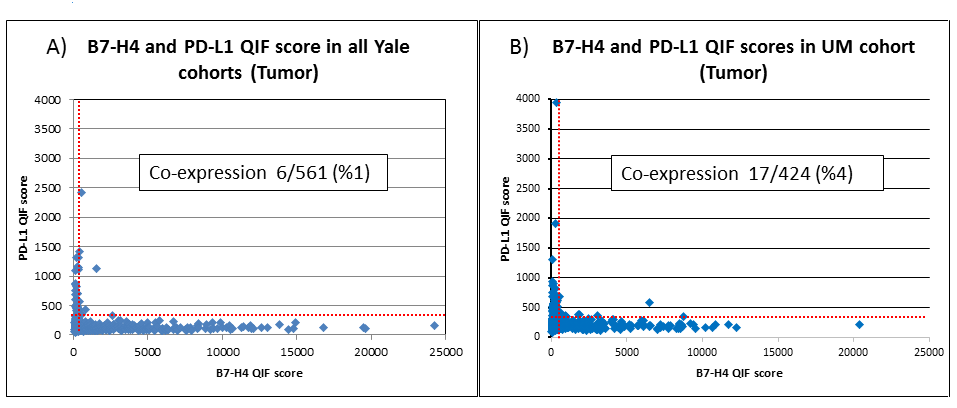
**

Concordance of B7-H4 and PD-L1 scatter plots for Yale (A) and UM (B) cohorts. Each case is represented by a blue dot. The dashed red line indicates the signal detection threshold determined by visual cut off for each marker. Co-expression rates are highlighted

**Supp Figure 3.** Scatter plots for TILs, PD-L1 and B7-H4 in Yale and UM Cohorts

**YALE COHORTS:**

**Supp. Figure 3. 1.** Yale Cohort TILs vs PD-L1 tumor (A) and stroma (B) scatter plots

**Supp. Figure 3. 2**. Yale Cohort TILs vs B7-H4 tumor (A) and stroma (B) scatter plots

**Supp. Figure 3.3.** Yale Cohort PD-L1 vs B7-H4 tumor (A) and stroma (B) scatter plots

**UM COHORT**

**Supp. Figure 3. 4**. UM Cohort TILs vs PD-L1 tumor (A) and stroma (B) scatter plots

**Supp. Figure 3.5**. UM Cohort TILs vs B7-H4 tumor (A) and stroma (B) scatter plots

**Supp. Figure 3.6.** UM Cohort PD-L1 vs B7-H4 tumor (A) and stroma (B) scatter plots

**Supp. Figure 4.** B7-H4 antibody validation


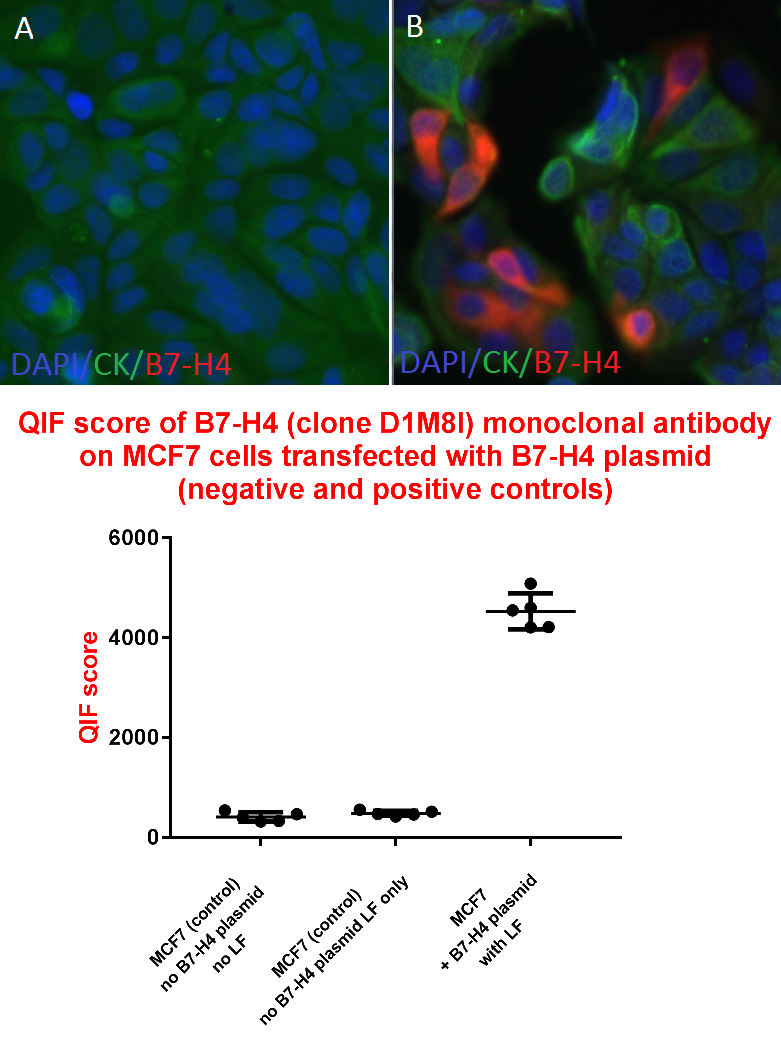


Representative fluorescence images showing the B7-H4 plasmid transfected cell lines A) negative control, B) positive control; below QIF score distribution of highest 5 Fields of view for the negative controls and plasmid transfected cell lines, bars are highlighting the mean with standard deviation

References:

1. Tringler B, Zhuo S, Pilkington G, et al. B7-h4 is highly expressed in ductal and lobular breast cancer. Clinical cancer research : an official journal of the American Association for Cancer Research 2005;11:1842-8.

2. Sun Y, Wang Y, Zhao J, et al. B7-H3 and B7-H4 expression in non-small-cell lung cancer. Lung cancer (Amsterdam, Netherlands) 2006;53:143-51.

3. Quandt D, Fiedler E, Boettcher D, Marsch W, Seliger B. B7-h4 expression in human melanoma: its association with patients' survival and antitumor immune response. Clinical cancer research : an official journal of the American Association for Cancer Research 2011;17:3100-11.

4. Liang L, Jiang Y, Chen JS, et al. B7-H4 expression in ovarian serous carcinoma: a study of 306 cases. Human pathology 2016;57:1-6.

5. Huang H, Li C, Ren G. Clinical significance of the B7-H4 as a novel prognostic marker in breast cancer. Gene 2017;623:24-8.
